# Supplementary material for: Acute Kidney Injury in Hospitalized Patients with COVID-19: Risk Factors and Serum Biomarkers
Source: Biomedicines. 2023 Apr 23;11(5):1246. doi: 10.3390/biomedicines11051246 (PMC10215395; doi:10.3390/biomedicines11051246)
Supplement: Supplementary file 1 [file biomedicines-11-01246-s001.zip › Supplementary Table S1.pdf]

Supplementary Table S1. Clinical and demographic characteristics of hospitalized patients with COVID-19 in the special sample (n = 89).

Parameters	General cohort (n=89)	AKI group (n=47)	No AKI group (n=42)	P-Value
Age, years	76 [66;84]	79 [67;84]	73.5 [64;82]	0.068
Men, n (%)	47 (52.8%)	25 (53.2%)	22 (52.4%)	0.939
Comorbidities:				
AH, n (%)	72 (80.9%)	42 (89.4%)	30 (71.4%)	0.033
DM, n (%)	24 (27%)	16 (34%)	8 (19%)	0.114
History of MI, n (%)	15 (16.9%)	10 (21.3%)	5 (11.9%)	0.241
AF, n (%)	20 (22.5%)	16 (34%)	4 (9.5%)	0.006
CKD C3-4, n (%)	29 (32.6%)	19 (40.4%)	10 (23.8%)	0.112
CHD III-IV FC, n (%)	16 (18%)	12 (25.5%)	4 (9.5%)	0.051
BMI, kg/m2	28.1 [25.7;31.6]	28.6 [25.9;31.5]	27 [25.2;32.1]	0.235
Obesity, n (%)	28 (31.5%)	15 (31.9%)	13 (31%)	0.702
Maximum area of lung damage on chest CT:				
less than 25%, n (%)	9 (10.1%)	5 (10.6%)	4 (9.5%)	
25-50%, n (%)	30 (33.7%)	13 (27.7%)	17 (40.5%)	0.054
51-75%, n (%)	31 (34.8%)	13 (27.7%)	18 (42.9%)	
more than 75%, n (%)	19 (21.3%)	16 (34%)	3 (7.1%)	
Oxygen saturation on admission, %	94 [92;96]	94 [90;95]	95 [92.75;96.3]	0.011
AKI, n (%)	47 (52.8%)			
stage 1, n (%)		29 (61.7%)		
stage 2, n (%)		11 (23.4%)		
stage 3, n (%)		7 (14.9%)		
Respiratory support				
Oxygen insufflation, n (%)	35 (39.3%)	20 (42.6%)	15 (35.7%)	
Mechanical ventilation:				0.0001
non-invasive, n (%)	4 (4.5%)	4 (8.5%)		
invasive, n (%)	19 (21.3%)	16 (34%)	3 (7.1%)	
Glucocorticoids, n (%),	83 (93.3%)	44 (93.6%)	39 (92.9%)	0.887
Antibiotics, n (%)	59 (66.3%)	39 (83%)	20 (47.6%)	0.0001
Immunobiological drugs (tocilizumab, olokizumab, levilimab), n (%)	32 (36%)	25 (53.2%)	7 (16.7%)	0.0001
Diuretics, n (%)	26 (29.2%)	20 (42.6%)	6 (14.3%)	0.004
iACE/ARB, n (%)	43 (48.3%)	19 (40.4%)	24 (57.1%)	0.092
Inotropes, n (%)	14 (15.7%)	13 (27.7%)	1 (2.4%)	0.001
ICU admission, n (%)	27 (30.3%)	22 (46.8%)	5 (11.9%)	0.0001
Outcomes:				
discharged, n (%)	54 (60.7%)	19 (40.4%)	35 (83.3%)	0.0001
died, n (%)	35 (39.3%)	28 (59.6%)	7 (16.7%)	
Duration of hospitalization, days	13 [10;18]	16 [11;21]	12 [10;15.3]	0.004
