# Supplementary material for: Acute Kidney Injury in Hospitalized Patients with COVID-19: Risk Factors and Serum Biomarkers
Source: Biomedicines. 2023 Apr 23;11(5):1246. doi: 10.3390/biomedicines11051246 (PMC10215395; doi:10.3390/biomedicines11051246)
Supplement: Supplementary file 1 [file biomedicines-11-01246-s001.zip › Supplementary Table S2.pdf]

Supplementary Table S2. Laboratory parameters of hospitalized patients with COVID-19 (n=500).

Parameters	General cohort (n=500)	AKI group (n=190)	No AKI group (n=310)	p-Value
On admission:				
Lymphocytes, 10^9/L	1.1 [0.7;1.4]	0.97 [0.7;1.4]	1.1 [0.7;1.4]	0.201
WBC, 10^9/L	5.9 [4.1;7.9]	6.4 [4.4;8.9]	5.5 [3.9;7.3]	0.001
PLT, 10^9/L	188 [147;244]	168 [130;230]	197 [158;251]	0.0001
CRP, mg/L	60.7 [25.6;103.6]	67.3 [31.9;108.3]	55.6 [24;9.53]	0.078
Ferritin, mcg/L	326.5 [185;543.2]	328 [177.5;586.6]	321.9 [186.9;496.9]	0.584
Glucose, mmol/L	6.5 [5.4;7.9]	6.6 [5.5;7.9]	6.4 [5.4;7.8]	0.399
LDH, U/L	505 [403.5;651.5]	509 [401.5;671.5]	505 [405.3;646.8]	0.77
Creatinine, mcmol/L	99 [85;120]	121 [102.2;143.8]	90 [81;103]	0.0001
Fibrinogen, mg/mL	5.5 [4.6;6.9]	5.4 [4.5;6.7]	5.7 [4.7;7.3]	0.093
D-dimer, mcg/mL	0.65 [0.4;1.2]	0.75 [0.4;1.5]	0.6 [0.4;1]	0.069
Potassium, mmol/L	4.4 [3.9;4.9]	4.4 [3.9;4.8]	4.4 [3.9;4.9]	0.15
Sodium, mmol/L	138.6 [136;141]	138 [135;140]	139 [136;142]	0.003
The worst parameters:				
Lymphocytes, 10^9/L	0.7 [0.5;1]	0.6 [0.3;0.9]	0.73 [0.6;1.1]	0.0001
PLT, 10^9/L	170 [127;227]	139.5 [98.8;183.8]	187 [147;242.3]	0.0001
CRP, mg/L	82.35 [45.7;132.7]	99.9 [67.6;179.6]	74.6 [35.8;119.5]	0.0001
Ferritin, mcg/L	436 [235.7;657]	543.2 [302;847.1]	387.6 [206.4;615.7]	0.0001
LDH, U/L	598 [446;806.5]	692 [482;1075]	556.5 [434;711.8]	0.0001
Creatinine, mcmol/L	104 [89;129.9]	137 [118.8;180.3]	92.7 [83;104]	0.0001
Potassium, mmol/L	4.15 [3.7;4.6]	4 [3.5;4.4]	4.3 [3.9;4.7]	0.0001
Sodium, mmol/L	138 [135;140.1]	137 [133.8;139.5]	138 [135.5;141]	0.0001
Fibrinogen, mg/mL	3.7 [2.8;4.9]	3.2 [2;4.2]	4 [3.2;5.2]	0.0001
D-dimer, mcg/mL	0.9 [0.5;2.2]	1.5 [0.6;4.9]	0.7 [0.4;1.3]	0.0001
Procalcitonin, ng/mL	0.1 [0.1;0.5]	0.3 [0.1;1.2]	0.1 [0.0;0.2]	0.0001
Proteinuria, %	162 (32.4%)	71 (37.4%)	91 (29.4%)	0.021
